# There’s a creepy guy on the other end at Google!: engaging middle school students in a drawing activity to elicit their mental models of Google

**DOI:** 10.1007/s10791-017-9306-x

**Published:** 2017-05-05

**Authors:** Christie Kodama, Beth St. Jean, Mega Subramaniam, Natalie Greene Taylor

**Affiliations:** 1 College of Information Studies, University of Maryland, College Park, MD 20742-4325 USA; 2 School of Information, University of South Florida, Tampa, FL 33620 USA

**Keywords:** Youth information seeking, Information retrieval, Mental models, Internet searching, Adolescents, Digital youth

## Abstract

Although youth are increasingly going online to fulfill their needs for information, many youth struggle with information and digital literacy skills, such as the abilities to conduct a search and assess the credibility of online information. Ideally, these skills encompass an accurate and comprehensive understanding of the ways in which a system, such as a Web search engine, functions. In order to investigate youths’ conceptions of the Google search engine, a drawing activity was conducted with 26 *HackHealth* after-school program participants to elicit their mental models of Google. The findings revealed that many participants personified Google and emphasized anthropomorphic elements, computing equipment, and/or connections (such as cables, satellites and antennas) in their drawings. Far fewer participants focused their drawings on the actual Google interface or on computer code. Overall, their drawings suggest a limited understanding of Google and the ways in which it actually works. However, an understanding of youths’ conceptions of Google can enable educators to better tailor their digital literacy instruction efforts and can inform search engine developers and search engine interface designers in making the inner workings of the engine more transparent and their output more trustworthy to young users. With a better understanding of how Google works, young users will be better able to construct effective queries, assess search results, and ultimately find relevant and trustworthy information that will be of use to them.

## Introduction

As youth are online with a frequency that increases every year—a 2015 study by the Pew Research Center (Lenhart 2015) found that nearly all teens report going online daily and one-fourth say they are online “almost constantly”—it is easy to think that young people are savvy Internet users in every way. Research shows, however, that most youth struggle with information literacy skills, such as finding credible resources and knowing the correct search terms to enter (Eastin 2008; Flanagin and Metzger 2008; Subramaniam et al. 2015a). When the information they seek is complicated, as it is when health information is the subject of a search, these struggles can intensify (Fergie et al. 2013; Hirsh 2004; Skopelja et al. 2008). Additionally, Agosto (2002a) finds that young people engage in satisficing behaviors, using techniques of reduction (such as returning to websites with which they are already familiar) based on web result page summaries, regardless of whether the page is actually the most relevant to the question. In their study on how middle- and high-school-aged students are able to distinguish and choose search engine results based on discipline and lexical relationship to the search topic, Keil and Kominsky (2013) found “a strong developmental shift during adolescence in evaluations of search engine results,” and, in particular, a marked improvement in the older students’ “ability to recognize deeper discipline-based relationships in the absence of lexical similarity” (p. 4). In another study (Rouet et al. 2011), researchers analyzed results from 174 7th–12th graders and found that “up to early secondary school, students are highly sensitive to surface relevance cues when selecting website titles from lists,” and that upper case keyword letters tended to be especially persuasive (p. 212). This study confirmed an earlier model by Rouet, the TRACE (“Task-based Relevance Assessment and Content Extraction”) model, that shows “relevance-based reading requires a combination of superficial and deep semantic processing (p. 212). In a 2016 study on teens, health, and technology, Wartella et al. (2016) found that, among those surveyed who indicated they go online for health information, over half (58%) start their searches by Googling a topic. Once they have Googled, half usually choose the first site listed and only seek additional information if the first site was unclear. Finally, in a related study conducted by the authors of this paper, we found evidence suggesting that young people engage in positive hypothesis testing, letting their pre-existing knowledge (which may or may not be accurate) influence their search term formulation and their selection of sources from a search result list (St. Jean et al. 2015).

The confluence of several factors, including information and digital literacy challenges such as these, an intensified focus on research skills in K-12 education through the Common Core and other educational standards (e.g., the American Association of School Librarians’ *Standards for the 21st Century Learner*), the increasing interest that young people have in going online to meet their health information needs (Wartella et al. 2016), and a motivation to better understand best practices in health, digital, and information literacy instruction, led a team of University of Maryland researchers to develop an after-school program, *HackHealth*. The team partnered with school librarians at four mid-Atlantic middle schools in the United States to design and implement an 8- to 12-week after-school program to guide youth between the ages of 10 and 14 in researching health topics of personal interest. In the once-weekly program, researchers and librarians led activities that addressed participants’ gaps in knowledge about the search process and helped the youth produce individual artifacts (such as PowerPoint presentations, posters, and digital comics) that incorporated their newly-gained domain knowledge in regard to a particular health topic. The program was implemented with the assistance of a grant from the National Library of Medicine to use design-based research (an approach in which researchers co-design learning programs with educators (Collins et al. 2004) to create lessons and modules for school librarians to use in collaboration with science and health teachers in both the classroom and the library.

Throughout the program, the attendees participated in a variety of data collection activities, one of which explored more deeply how the participants view, understand, and conceptualize the Google search engine. Participants were asked to draw how they thought Google worked “behind the scenes” to find search results for user queries and to then explain their drawings. Google was selected as the search engine of choice for this activity as it is, by far, the most popular search engine among U. S. Internet users (eBizMBA, Inc. 2017) and as the majority of teens who go online for health information begin their searches with Google (Wartella et al. 2016). This particular data collection strategy was designed to better understand the knowledge the participants have of search engines, as well as the digital literacy instruction that they have, or have not, received. The researchers also wondered how these particular students’ perceptions aligned with the findings of prior researchers who have elicited users’ mental models of online search.

In the following section, an overview of the literature on mental models is provided. Next, the methods used to carry out this study are delineated. The results of the study are then reported, followed by a discussion of the results. The paper concludes with the limitations of this study and ideas for future research. This study extends prior research in understanding young people’s digital and information literacy skills with respect to interactions with online search engines. It also provides new understanding as to how educators can explain complicated search processes to young people.

## Literature review

The concept of a mental model has appeared in the literature of several different disciplines, including cognitive science; cognitive psychology; risk perception and communication; system dynamics; and human–computer interaction (Doyle and Ford 1998). The modern conceptualization of “mental model” dates back to Scottish psychologist Kenneth Craik (1943), who defined mental models as “small-scale models” of reality (Johnson-Laird 1989; Westbrook 2006). Studies of mental models have become increasingly prevalent in recent decades, resulting in many different definitions of the term (Doyle and Ford 1998; Johnson-Laird 1989). Doyle and Ford (1998) analyzed these varying definitions and proposed the following conceptual definition based on how the term was most commonly being used within the field of System Dynamics: “A mental model of a dynamic system is a relatively enduring and accessible, but limited, internal conceptual representation of an external system whose structure maintains the perceived structure of that system” (p. 17). A more broadly applicable definition of mental models is offered by Besnard et al. (2004): “simplified, cognitively acceptable versions of a too-complex reality” (p. 119).

Donald Norman (1983), who subsequently coined the highly relevant term/philosophy “user-centered design,” adapted the term “mental model” for the field of human–computer interaction. His definition was based on his experiences observing people interacting not only with computers, but also calculators, digital watches and cameras, video cameras and recorders, and aircraft instrumentation. Norman described mental models as people’s continuously evolving cognitive representations of a system that incorporate their beliefs regarding the way the system works. Norman emphasized that a person’s mental model of a particular system both guides his/her use of the system and is iteratively (re)informed by his/her interactions with the system across time.

Norman (1983) distinguished between a conceptual model and a mental model—a conceptual model is an accurate and comprehensive representation held by the system designer, while a mental model is an often incomplete, limited, unstable, unscientific, and parsimonious representation held by the user. Ideally, the system designer’s conceptual model of the system is adequately reflected in the system design in such a way that the user is then able to construct a corresponding mental model; however, in reality, users’ actual mental models are often “messy, sloppy, incomplete, and indistinct structures” (Norman 1983, p. 14). In fact, Norman concludes, “Most people’s understanding of the devices they interact with is surprisingly meager, imprecisely specified, and full of inconsistencies, gaps, and idiosyncratic quirks” (p. 8). These deficiencies have been posited to arise from both bounded rationality (Simon 1956), which holds that people are rational within limits, often preferring to settle for a less optimal solution that is less costly to identify and enact (“satisfice”), and a person’s limited previous experience with a system (Doyle and Ford 1998). Researchers have found that people may use mental models that are comfortable and familiar to them, without regard for their actual efficacy (Brandt 2001). Furthermore, a random coupling of events unfolding in sequence can serve to reinforce an incorrect mental model, particularly as people tend to look for evidence that confirms what they already believe (a phenomenon known as “confirmation bias”) (Besnard et al. 2004; Klayman and Ha 1987, 1989).

A person’s mental model of a system can be used to predict and explain the ways in which he/she interacts with the system (Norman 1983). Studies seeking to elicit people’s mental models have drawn on a range of data collection techniques, including direct observation of people interacting with a device and thinking aloud (e.g., Holman 2011; Li 2007; Norman 1983), interviews (e.g., Brandt and Uden 2003; Li 2007; Yan 2005; Zhang 2008a, b), and asking people to draw their understanding of a particular concept, process, or system (e.g., Denham 1993; Dinet and Kitajima 2011; Gray 1990; Papastergiou 2005; Rieh et al. 2010; Thatcher and Greyling 1998; Westbrook 2006). The process of eliciting mental models through drawing has, in fact, not only been used as a data collection method for research purposes, but also as an instructional method by teachers who wish to learn about their students’ understandings of a science concept and how these understandings evolve across time, while simultaneously facilitating their building of mental models through the process of drawing itself (Glynn 1997). A person’s mental model can reveal his/her developing understanding of a system, along with any misconceptions he/she might hold. This information, in turn, can help a teacher (or librarian) provide better-tailored lessons for particular student(s) at a particular point in time (Brandt 2001; Denham 1993; Glynn 1997; Papastergiou 2005).

Although mental models can open a window into the way in which people understand a process or system, many researchers have questioned whether the view we obtain in this way is, in fact, accurate. A number of limitations of mental model research have been noted by researchers across various disciplines. Most fundamentally, it is impossible to directly observe people’s mental models (Doyle and Ford 1998; Richardson et al. 1994) and people may not be sufficiently self-aware to be able to verbalize their mental models (Westbrook 2006). The latter is likely especially true for children, who may also have insufficient manual dexterity skills and/or mental agility to prepare an accurate representation of their mental models (Marhan et al. 2012). Goodman (1976) further pointed out that some children’s drawings are merely symbolic, failing to resemble the drawn item and to convey any detailed information about it.

People’s mental models may be inaccurate for a variety of reasons, including a person’s lack of awareness of all relevant belief structures at the same time (Norman 1983) and a researcher’s vague or ambiguous instructions and questions and/or his/her misinterpretation of study participants’ responses, particularly when the participants are children (Panagiotaki et al. 2009). Another reason that people’s mental models may be inaccurate can be linked to the demand characteristics inherent in the study situation (Norman 1983). That is, people may feel pressured to come up with “something” simply because they’ve been asked to do so. Or they may try to come up with something they believe the researcher would like to see/hear (Norman 1983; Richardson et al. 1994). Furthermore, the very process of asking people to draw mental models can result in changes to their mental models (Doyle and Ford 1998; Richardson et al. 1994). Richardson and colleagues (1994) term this the “mental model uncertainty principle,” noting that “mental models are not directly accessible or observable; efforts to elicit mental models distort what is elicited” (p. 2).

Despite these limitations, many researchers across several different fields have conducted studies in which they have sought to elicit people’s mental models. Several studies have employed drawing as a technique to elicit people’s mental models, in contrast to studies that have used more closed-ended processes, such as asking participants to select from a set of already-existing images or metaphors (see, for example, Palmquist 2001). Some studies that have used an open-ended drawing technique to elicit people’s mental models of various processes and systems are briefly reviewed here. The studies covered here focus mainly on those eliciting people’s mental models of information retrieval systems and processes, although studies have used similar methods to elicit other types of mental models, such as violin students’ (aged 8–18) perceptions of the teacher-student partnership, their violin lessons, and the outcomes they had experienced (Creech and Hallam 2006); children’s (aged 8–12) perceptions of school bullying and victimization (Bosacki et al. 2006); and incoming university freshmen’s conceptualizations of the greenhouse effect (Libarkin et al. 2015).

Westbrook (2006) elicited graduate students’ (aged 23–29) mental models of the information-seeking process for a research paper by asking them to sketch a diagram of their process and to write a detailed description of their diagrams. Rieh et al. (2010) elicited undergraduate students’ (aged 18–25) mental models of their university’s institutional repository (IR) by pairing students and asking each student in the pair to conduct two search tasks using both Google and the IR, and to then draw how they thought the IR worked and explain their drawing to their teammate. Holman (2011) elicited first-year University students’ (aged 16–19) mental models of search engines and article databases by asking them to describe how they thought these tools returned results based on the terms they had input and to draw or diagram the connections between the terms they input and their relationships to the results that were retrieved. Thatcher and Greyling (1998) elicited University respondents’ (including undergraduate students, postgraduates, academic staff, and administrative staff, but predominantly undergraduate students) mental models of the Internet by asking them to draw and annotate a sketch of how they thought the Internet is structured and how they thought it worked. These respondents were also asked to prepare a 1-paragraph explanation of their drawings. Zhang (2008a, b) elicited undergraduate students’ (aged 17–22) mental models of the Web by asking them to “draw a diagram or a picture of their perceptions about the Web” (Zhang 2008b, p. 2090) and to then describe their drawings.

The vast majority of studies that have sought to elicit people’s mental models through a drawing activity have focused on undergraduate and/or graduate students (Holman 2011; Rieh et al. 2010; Westbrook 2006; Zhang 2008a, b); however, some have focused on children (Denham 1993; Dinet and Kitajima 2011; Papastergiou 2005; Yan 2005, 2006, 2009). Using a technique similar to Zhang’s (2008a, b) work with undergraduates, Dinet and Kitajima (2011) elicited French children’s (aged 10–14) mental models of the Web by asking them to draw a picture of their perceptions of the Web. Papastergiou (2005) elicited Greek high school students’ (aged 12–16) mental models of the Internet, asking them to prepare an annotated drawing of the Internet, along with an accompanying written explanation of their drawing. Yan (2005, 2006, 2009) elicited children’s (ages 5 through 12) and adults’ mental models of the Internet by asking them to draw two pictures of “what the Internet looks like” (Yan 2005, p. 390). Denham (1993) elicited children’s (aged 9–14) mental models of computers by asking them to draw what they would expect to see if they could shrink themselves to a size so tiny they could fit into the rear port of a micro-computer and climb inside. Several studies that have used drawing activities such as these have confirmed the usefulness of this technique to uncover people’s mental models (Devine-Wright and Devine-Wright 2009; Zhang 2008b), particularly when working with children (Dinet and Kitajima 2011). Underscoring the relative advantages of engaging children in a drawing activity, one study (Harrison et al. 2007) found that young children’s (6-year-olds) drawings of themselves with their teacher at school provided a better indicator of the emotional quality of the child-teacher relationship than the children’s verbal responses to questions directly aiming to elicit their feelings about their teacher.

Many researchers who have conducted studies to elicit people’s mental models have devised a typology to categorize the drawings prepared by their participants. One of the most comprehensive categorization schemes was developed by Papastergiou (2005) to classify her participants’ drawings of the Internet: (1) Non-digital entity (very simplistic drawing in which no computer was portrayed); (2) Services and content (drawing focused on the types of services or information on the Web and no computer was drawn); (3) User’s computer (drawing of just the user’s computer); (4) Huge remote computer (drawing of a computer that does not belong to the user, with no accompanying connections); (5) Connection between two computers (drawing of two connected computers); (6) Few computers linked through a connection point (drawing that shows two to four computers communicating by linking to a central connection point); (7) Computer network (drawing that shows a large network of connected computers); and (8) Network of computer networks [drawing that shows a very large network of computers with a hierarchical structure, depicting the Internet as many networks, many central computers (servers), and many user computers (nodes)].

Another very relevant categorization scheme was devised by Dinet and Kitajima (2011) to categorize their child participants’ drawings of the Web. They identified six categories of drawings, the first four of which had been previously identified by Zhang (2008a): (1) Technical view (drawing shows a group of computers, servers, modems, and CPUs); (2) Functional view (drawing shows a place to shop, play games, e-mail, pay bills, look for information, and do research); (3) Process view or search engine-centered view (drawing shows search engines as the center of the Web); (4) Connection view (drawing shows worldwide connections between information, people, computers, mobile phones, and web pages); (5) Techni-functional view [a combination of (1) and (2)]; and (6) Functional-connection view [a combination of (2) and (4)].

Thatcher and Greyling (1998) devised the following categorization scheme to categorize their adult participants’ drawings of the Internet: (1) Interface and utilitarian functionality (focus on applications or types of information available); (2) Central database (focus on central computer with several connected users); (3) User to the world (focus on one’s own computer as being connected to a larger world); (4) Simple connectivity (focus on multiple computers linked together); (5) Simple modularity (focus on computers connected together in a network); and (6) Modularity and networking [focus on structural elements of the Internet, often transmission methods (e.g., IP addresses) and transmission devices (e.g., satellites or modems)].

The findings from these studies revealed that many participants’ mental models exhibited an incomplete understanding (e.g., Holman 2011; Papastergiou 2005; Rieh et al. 2010; Yan 2005, 2006, 2009; Zhang 2008a, b). Papastergiou (2005), for example, found that just seven of her 340 high school student participants created a drawing of the Internet that fit into her most sophisticated category—“network of computer networks.” She indicated that students’ drawings rarely portrayed the structural underpinnings of the Internet, and tended to focus mostly on the user’s computer and what he/she sees on the screen. Furthermore, several researchers found that older children (Denham 1993; Dinet and Kitajima 2011; Yan 2005, 2006, 2009) and/or those who were more experienced in using the Internet (Thatcher and Greyling 1998; Yan 2005, 2009) tended to have more complete mental models. For example, Denham (1993) found that her older participants’ (11–12 year-olds) drawings were more likely than those of her younger participants (9–10 year-olds) to demonstrate an awareness of the hidden processes between computer input and output. Dinet and Kitajima (2011) found that their older participants’ (13–14 year-olds) drawings were more likely than those of their younger participants (10–11 year-olds) to incorporate a view of the Web that went beyond just the process of using it and encompassed the actual functions for which one might make use of it. Yan (2005, 2006, 2009) found that his older participants’ (11–12 year-olds) drawings demonstrated a greater understanding of the “technical and social complexity of the Internet” (Yan 2005, p. 393) than those of his younger participants (5–8 year-olds and 9–10 year-olds).

Underscoring the importance of mental models, some researchers have found a correlation between participants’ mental models of a system and their performance using the system. Gray (1990), for example, found that adult users of a hypertext navigation system (i.e., Guide) who held a linear mental model of the system tended to encounter greater navigation difficulties. More recently, Dinet and Kitajima (2011), issued a call for more research into users’ mental models because they similarly found that their child participants’ (aged 10–14) understandings of the Web (i.e., their mental models) affected how well they were able to make use of it.

In summary, studies of users’ mental models have become increasingly prevalent over the past 30 years or so and drawing, in particular, has been used to elicit adults’ and children’s mental models for both research and instructional purposes. Although several potential limitations have been associated with the processes used to elicit people’s mental models, particularly those of children, many researchers have reached important findings utilizing this method. In this paper, the results from an investigation into the mental models of Google held by 26 middle school students (ages 10–14) participating in the *HackHealth* after-school program are reported. To the best of our knowledge, our study represents the first investigation that sought to elicit the mental models of a search engine (Google, in particular) of middle school children. In the following section, the specific drawing activity that was used to elicit participants’ mental models for this investigation, along with the techniques used to analyze these drawings, are described in detail.

## Methods

In order to understand how middle school students perceive how Google works, the research team conducted an activity during one of our regular after-school *HackHealth* sessions in participants’ school libraries. Students were asked to represent how they think Google works by drawing a picture and/or writing down some words. The specific methods used for recruitment of students into the *HackHealth* program, as well as for data collection and analysis in relation to the drawing activity are described below.

### Recruitment

For the 2014–2015 academic year, a total of 33 students across four middle schools in the greater Washington D.C. metro area participated in *HackHealth*. Two of the four schools are Title I schools, indicating that at least 40% of the students have enrolled in the free and reduced-price meals (FARMS) program. At these two particular schools, 85–90% of the students are enrolled in this program (Maryland State Department of Education 2015). The school librarian at each of the four schools carried out the recruitment efforts using several different methods. They visited health classes to announce the program and gauge student interest, asked health and science teachers in their schools to provide referrals of students who would be interested and/or would be likely to benefit from participating in *HackHealth*, asked their school principals to make announcements regarding the program, and mentioned the program to students who came into the school library for other purposes.

### Student participants

A total of 26 students participated in this activity. A list of participants, along with their demographic information and their ratings of their ability to use the Internet, find the health information they need, assess the trustworthiness of health information, and apply health information within their own lives, is shown in Table 1. Information in Table 1 was collected through a health interest survey the project team conducted with each student during Week 1 of the *HackHealth* program.

**Table 1:** Participant demographic information and self-reported ability levels

No.	Demographic information						Self-reported ability levels			
	(1) Student (S##)	(2) M/F	(3) Race^a^	(4) Age	(5) School	(6) Grade	(7) Ability to Use the Internet^b^	(8) Ability to Find Needed Health Info.^c^	(9) Ability to Assess Trust-worthiness of Health Info.^d^	(10) Ability to Apply Health Info.^e^
1	S01	M	H	13	1	7	5	4	2	4
2	S02	M	A	11	1	5	4	5	4	4
3	S03	M	A	10	1	5	5	3	5	4
4	S04	M	H	11	1	5	5	5	3	5
5	S05	M	B	13	1	7	3	3	2	3
6	S06	F	B	13	2	7	5	3	2	4
7	S07	F	H		2	7	5	5	5	5
8	S08	F	A	14	2	8	5	4	5	4
9	S09	F		13	2	8	3	2	2	3
10	S10	M	C	14	2	7	5	2	0	2
11	S11	F	B	14	2	8	5	3	1	3
12	S12	F	B	13	2	7	5	3	2	4
13	S13	F	B	14	2	8	5	2	1	1
14	S14	M	AI; B		3	7	4	2	2	2
15	S15	F	B	12	3	6	5	3	3	4
16	S16	M			3					
17	S17	M			3	8	5	3	2	3
18	S18	F			3					
19	S19	F	H	13	4	8	4	3	4	2
20	S20	M	H	12	4	7	5	3	1	5
21	S21	M	H	12	4	7	5	3	5	4
22	S22	F	H	12	4	7	4	2	0	1
23	S23	F	H	13	4	7	4	2	2	2
24	S24	F	H	13	4	7	4	2	2	3
25	S25	F	B	12	4	7	5	4	2	3
26	S26	M	B	12	4	7	4	2	1	2

^a^ *A* Asian, *AI* American Indian or Native American, *B* Black or African-American, *C* Caribbean, *H* Hispanic or Latino

^b^The survey question stated, “How good are you at using the Internet?” and students responded using a 5-point scale: 1 (not good at all); 2 (not very good); 3 (OK); 4 (pretty good); and 5 (very good)

^c^The survey question stated, “How good are you at finding the health information you need?” and students responded using a 6-point scale: 0 (no experience); 1 (poor); 2 (fair); 3 (good); 4 (very good); 5 (excellent)

^d^The survey question stated, “When you find health-related information, how well can you tell if it can be trusted or not?” and students responded using a 6-point scale: 0 (no experience); 1 (poor); 2 (fair); 3 (good); 4 (very good); 5 (excellent)

^e^The survey question stated, “How good are you at applying health-related information to your own life?” and students responded using a 6-point scale: 0 (no experience); 1 (poor); 2 (fair); 3 (good); 4 (very good); 5 (excellent)

Twelve boys (46%) and 14 girls (54%) participated in this activity. They ranged in age from 10 to 14 and were in grades 5–8. However, the majority (n = 15; 65%) were between the ages of 12 and 13. All of the student participants reported having access to the Internet at home, either through a personal computer, tablet, or smartphone. The seven students who did not complete this activity were absent during this particular *HackHealth* session and, therefore, are not included in the analysis of these drawings.

### Compliance with ethical standards

Approval for the *HackHealth* program and the associated research activities was secured from both the researchers’ University IRB, as well as the Department of Research and Evaluation in the public school system where *HackHealth* was conducted, before the program was offered to students. Each student who chose to participate in *HackHealth* attended an information session in their school’s library a week prior to the first session of the after-school program. Students were asked to read and sign a consent form that introduced the research team, gave a brief overview of the 12-week program, and described how the student’s privacy would be protected if they decided to participate. Participating students were also given a consent form to take home for their parents to read and sign, indicating their agreement that their child could participate in the *HackHealth* after-school program. Consent forms were translated into Spanish for those families who needed it. Signed consent forms from both students and parents were collected from the students at the beginning of the first *HackHealth* session.

### Data collection

During the third or fourth week of the *HackHealth* program at each school, the researchers conducted an activity with students to elicit their perceptions regarding how they think Google works. The researchers provided each student with a large piece of white construction paper and markers, and verbally asked students to draw a picture or write down in words how they think Google works “behind the scenes” to find websites for people and to then verbally describe their drawings. The instructions given were kept brief and very open-ended, as some researchers (e.g., Barrett and Bridson 1983; Light 1985) have found that the verbal instructions given to child study participants can systematically influence their resultant drawings. Students worked independently on their drawings and were given approximately 20 min to work on their ideas. After all the students completed their drawings, each participant was given 1–2 min to share and describe their drawings to those present, including the school librarian, other *HackHealth* participants, and members of the *HackHealth* research team. Following each student’s presentation, we drew on cues present in his/her drawing and employed interactive questioning in order to obtain a deeper and more comprehensive understanding of the student’s drawing. This process of combining a drawing activity and interactive questioning has been found to facilitate children’s recall (Barlow et al. 2011). All student presentations and responses to our inquiries were audio-recorded and subsequently transcribed for future comparison and analysis with their actual drawings.

### Data analysis

In total, 26 unique students prepared a drawing for this activity. To facilitate data analysis across all four members of the research team, one researcher took a digital picture of each drawing, which was then subsequently stored on a password-protected data repository so that all members of the research team could access the drawings with ease. A thematic content analysis approach similar to that described by Libarkin et al. (2015) was then employed. Using the digital copies of the participants’ drawings and the transcriptions of their accompanying verbal descriptions, each researcher independently viewed each student’s drawing and performed an initial round of open coding, inductively coming up with words or phrases that they felt described what they saw in the picture and what they heard in the participant’s explanation. To facilitate and organize the data analysis, one researcher created a spreadsheet template for each researcher to fill out that included all the students’ names, pseudonyms, school names, transcriptions of student explanations of their drawings, and a column for the individual researcher’s codes.

During this first phase of coding, the research team members also independently created and assigned higher-level typologies to each of the student drawings. This initial round of coding was done individually and independently to ensure that researchers did not bias one another’s codings. As mentioned earlier, researchers in previous studies who investigated people’s mental models of the Internet created typologies to represent their findings in a clear and organized way. As none of these existing typologies accurately and comprehensively encompassed *HackHealth* participants’ drawings, the *HackHealth* researchers allowed the typology entries to emerge inductively as they were analyzing the participants’ drawings.

**Fig. 1:**
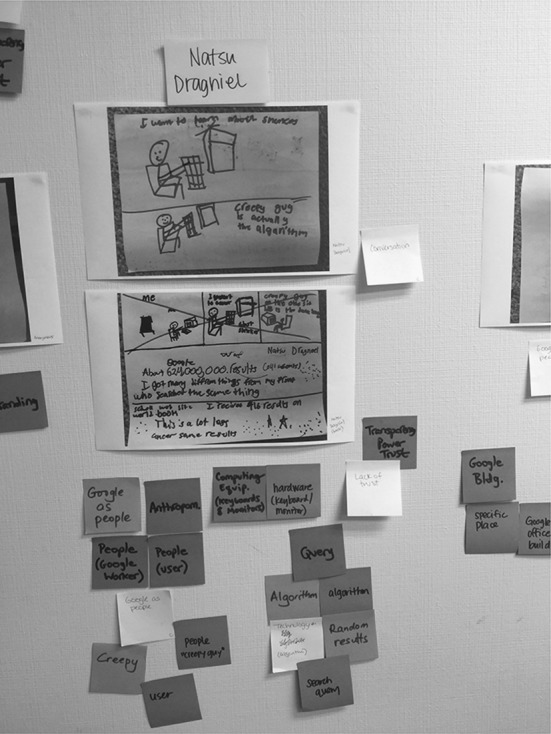
S17’s Team Coding

**Fig. 2:**
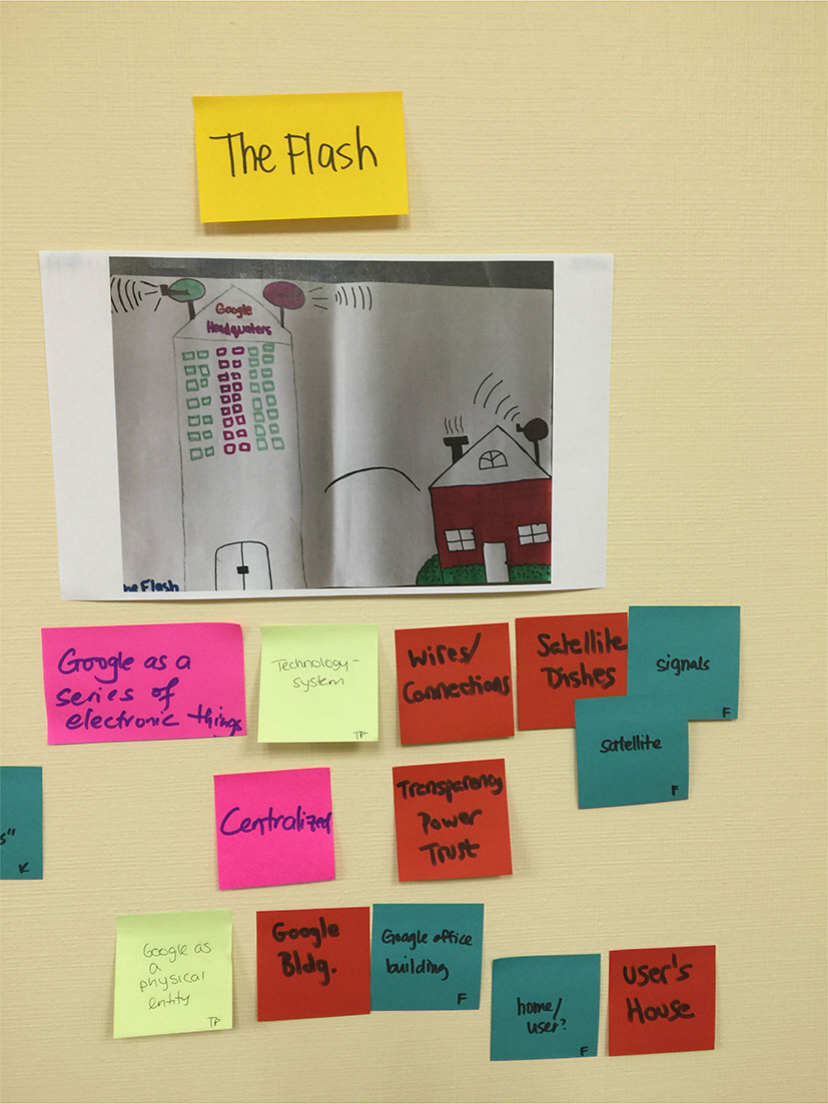
S26’s Team Coding

After each researcher viewed all of the drawings and created codes for each one, the research team met to compare and discuss their codes using a brainstorming process called “sticky noting” (Fails et al. 2012). “Sticky noting” is a participatory design technique useful for visualizing the similarities, differences, and connections between different individuals’ codes or descriptions. Sticky notes can be grouped together based on similarities, allowing themes to emerge that explain the similarities and differences in codes. To facilitate the “sticky noting” process, a member of the research team photocopied and taped all the mental model drawings on the walls. Each researcher chose a different color of sticky notes to write down their codes from the first round of individual analysis, one code per sticky note. After each researcher posted all their codes for each mental model under the corresponding drawing, the research team grouped similar codes for each individual student drawing. Digital pictures were captured of the resulting walls from the sticky note coding session and these pictures were then used to input all codes into a spreadsheet for future rounds of data analysis. Figures 1 and 2 show the team’s codings of S17’s and S26’s drawings, respectively.

During this initial group coding session, the research team also collectively developed and agreed upon a typology scheme consisting of six entries and a 14-item coding dictionary that each researcher would use to analyze each drawing during the second round of coding. The typology and coding dictionary were created based on a discussion of each researcher’s individual typology and code suggestions. The six-item typology that emerged from our initial inductive analysis of the drawings and from the students’ accompanying verbal descriptions, along with the researchers’ agreed-upon definitions, are shown in Table 2. The 14 codes that similarly emerged through the researchers’ analysis of the drawings and the students’ verbal descriptions, along with the researchers’ agreed-upon definitions are shown in Table 3.

**Table 2:** Typology of participants’ drawings

Typology Entry	Definition
Google as people	Drawings represent Google as a person or people, whether Google workers or scientists, who work on behalf of Google to find information for the user. Drawings also depict Google personified; objects that talk, think, or have other human characteristics
Google as equipment	Drawings have some form of computer hardware (such as monitor, keyboard, mouse, CPU, tablet, smartphone) as a main focus
Google as connections	Drawings focus on the connections that allow Google to work. Wires/cables connecting multiple computers together, a satellite/antenna transmitting signals, and the connection between a user and how the participant views Google are examples of drawings in this category
Google as a physical space	Drawings represent Google as a building (i.e., house, office building, Google headquarters, or office space)
Google as interface	Drawings show the Google interface and oftentimes a close depiction of the Google logo in color. Drawings include the features and functionality that Google has on its webpage, including the search box/bar, “I’m Feeling Lucky” button, page count, and amount of time the search took
Google as codes	Drawings depict Google as a series of codes, whether numeric, alphabetic, alphanumeric or something else. These drawings depict how the participant thinks Google works from a technological standpoint

**Table 3:** Final coding dictionary

Code	Definition
Anthropomorphism	Google is shown or described as being a person or doing something a person would normally do (like talk, walk, think, etc.)
Branding	Any image or reference to a specific company logo or brand (use of colors, font/type, etc.)
Computer code	Any kind of computer code, such as numbers or symbols
Computing equipment	Computer hardware is shown or described
Connections	Image or description that highlights connections between various types of computing equipment, including monitors, CPU’s, keyboards, etc. Also includes images/descriptions that feature an antenna or satellite dish
Features/functionality	Special capabilities of Google are shown or discussed
Gender balanced	Both males and females are shown or described
Google worker	Image shows Google worker(s) researching and returning the results
Intelligence	Google is shown or described as involving people and/or a system that is smart or knows a lot
Place	Image or description includes buildings and/or other types of physical space
Query	Image or description includes the actual words the user typed into the Google search bar
Transparency	Image or child’s description refers to transparency (or lack of transparency) as to how Google works
Trust	Image or child’s description expresses trust (or mistrust) toward Google
User	Image shows the user who needs information and is going to Google to find it

Two members of the research team then conducted a second round of coding using the aforementioned 14-item coding dictionary and 6-item typology scheme. These two researchers first independently coded four of the 26 mental model drawings (one randomly selected from each school). Intercoder reliability was then calculated using Scott’s Pi formula (Holsti 1969), yielding a figure of 88%. Finding this intercoder reliability rate sufficient, the two researchers then each coded half (11) of the remaining 22 drawings.

## Results

Student drawings were assigned to one (or more) of the six typology entries that researchers inductively developed and then finalized during the first round of group coding. All drawings were assigned a primary typology entry. Twenty-one (81%) drawings were assigned a secondary typology entry as well, and twelve (46%) of these were also assigned a tertiary typology entry. Table 4 shows the number and percentage of student drawings to which each entry in the typology was assigned. Each of these typologies is described in more detail, with relevant examples drawn from the pool of student drawings, in the subsections that follow.

**Table 4:** Counts (percentages) of drawings assigned to each mental model typology entry

Typology entry	Primary	Secondary	Tertiary	Sum
Google as people	10 (38.5%)	3 (11.5%)	1 (3.8%)	14 (53.8%)
Google as equipment	1 (3.8%)	8 (30.8%)	4 (15.4%)	13 (50.0%)
Google as connections	5 (19.2%)	4 (15.4%)	3 (11.5%)	12 (46.2%)
Google as a physical space	4 (15.4%)	4 (15.4%)	1 (3.8%)	9 (34.6%)
Google as an interface	3 (11.5%)	1 (3.8%)	3 (11.5%)	7 (26.9%)
Google as codes	3 (11.5%)	1 (3.8%)	0 (0.0%)	4 (15.4%)

### Google as people

“Google as people” was the most represented typology among all the drawings; 10 of the 26 (38%) representations were assigned a primary typology entry of “Google as people.” A total of 14 (54%) drawings were assigned to this typology entry overall. The drawings in this category all had some aspect that suggested the student conceived of Google as being a person or people (or like a person or people) working to find information for the user. These drawings focused on people—either people who are employed by Google working in a Google office building finding information for users or just people who give the information they find to Google.

**Fig. 3:**
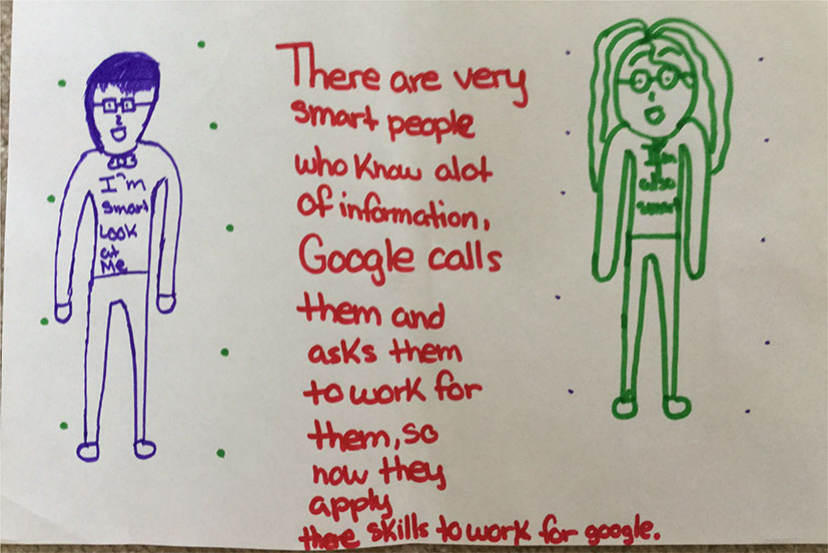
Drawing by S06

**Fig. 4:**
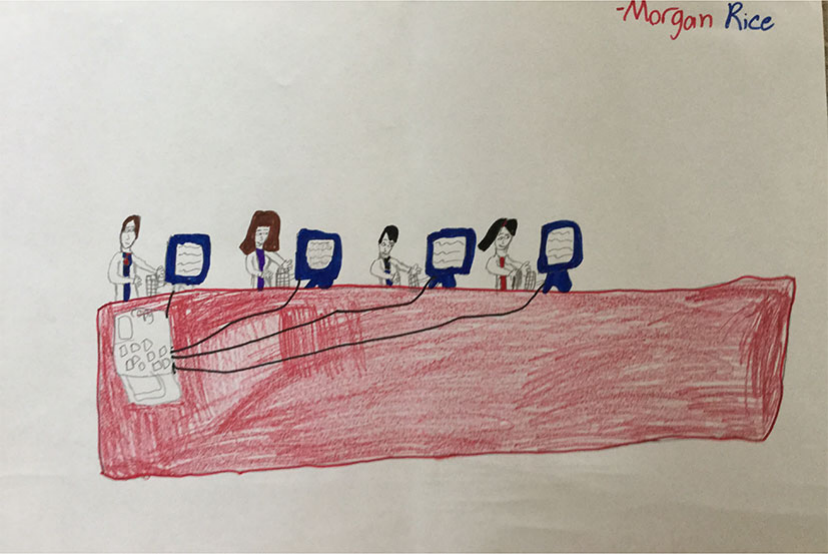
Drawing by S25

**Fig. 5:**
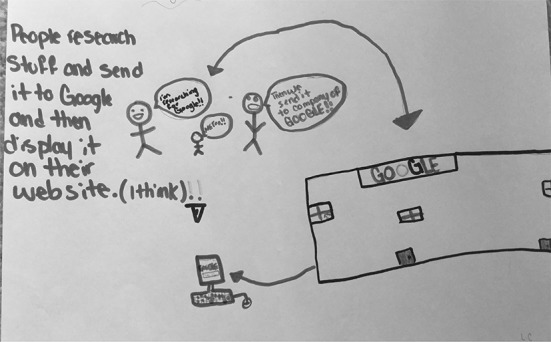
Drawing by S22

Figure 3 shows S06’s drawing of a man and a woman, whom she describes as “smart people who know a lot of information.” According to S06, these people are solicited by Google to work for them because they know a lot. In explaining her drawing, she described that she drew eyeglasses, ties, and t-shirt slogans to emphasize that they are smart people. In Fig. 4, S25 describes her mental model as “scientists at the Google answering scientific questions.” In these cases, the people who work for Google are shown as intelligent and in possession of a lot of information, which is then transferred to Google and in turn transferred to the users. In a similar vein, S22 (Fig. 5) said that Google works when “people research stuff and send it to Google” for them to post online.

**Fig. 6:**
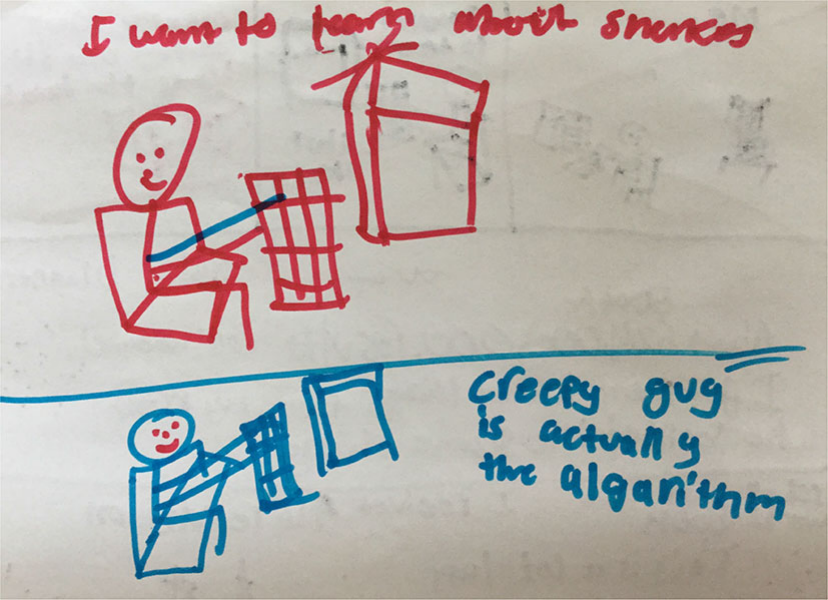
Drawing by S17

S17’s drawing (Fig. 6) shows the user who wants to learn about snakes and, instead of depicting Google as intelligent or as smart people, S17 described the “creepy guy on the other end at Google, who’s actually the algorithm” and who gives the user all the information about snakes.

**Fig. 7:**
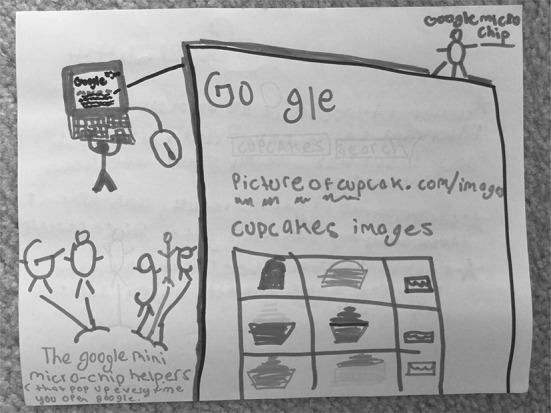
Drawing by S18

Although not necessarily depicted as human beings, S18 (Fig. 7) describes Google as having “micro-chip helpers” (like a “mini person” and reminiscent of the Microsoft paperclip character meme, Clippy, to the researchers) who help people find what they are looking for on the Internet. In S18’s depiction, Google is personified—her “micro-chip helpers” have arms and legs that can move around and help users find what they are searching for online.

### Google as equipment

**Fig. 8:**
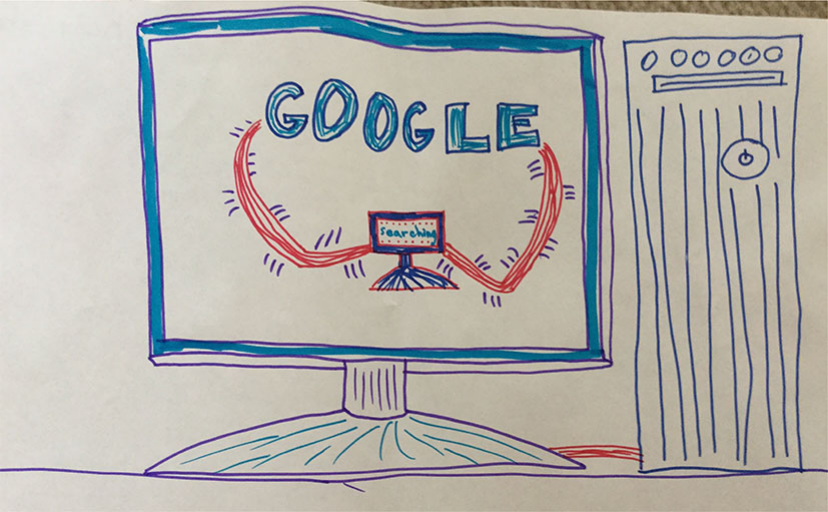
Drawing by S08

This typology entry, which was the second most commonly assigned by researchers, includes depictions of Google that focus on the computer equipment through which it is accessed. In describing her drawing of a CPU and a monitor showing Google conducting a search, S08 (Fig. 8) stated, “So this is how Google works. You type in the words and then the power would come from here and it will be going to the computer that will start searching and give you results.” The computer equipment is the main focus of her drawing; there is no mention or hints in her description or in her drawing to suggest aspects of any of the other typology entries.

**Fig. 9:**
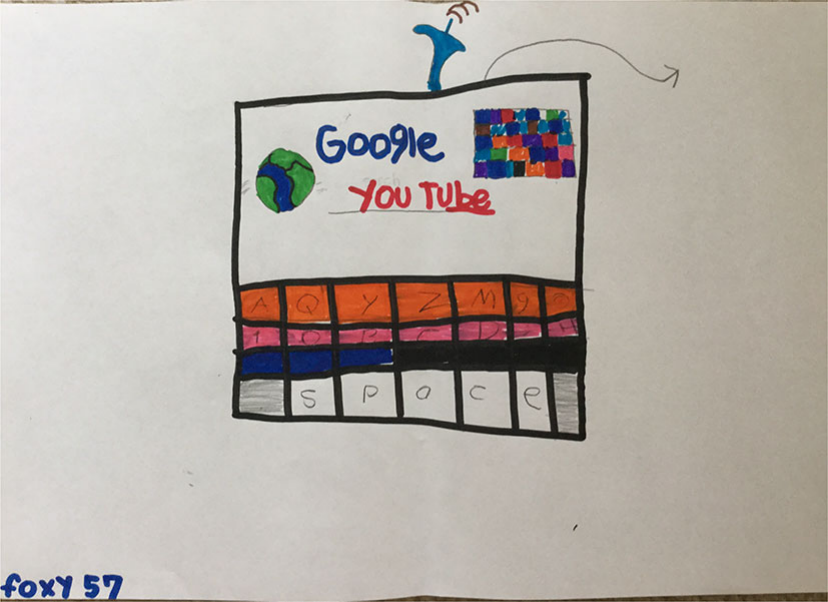
Drawing by S21

**Fig. 10:**
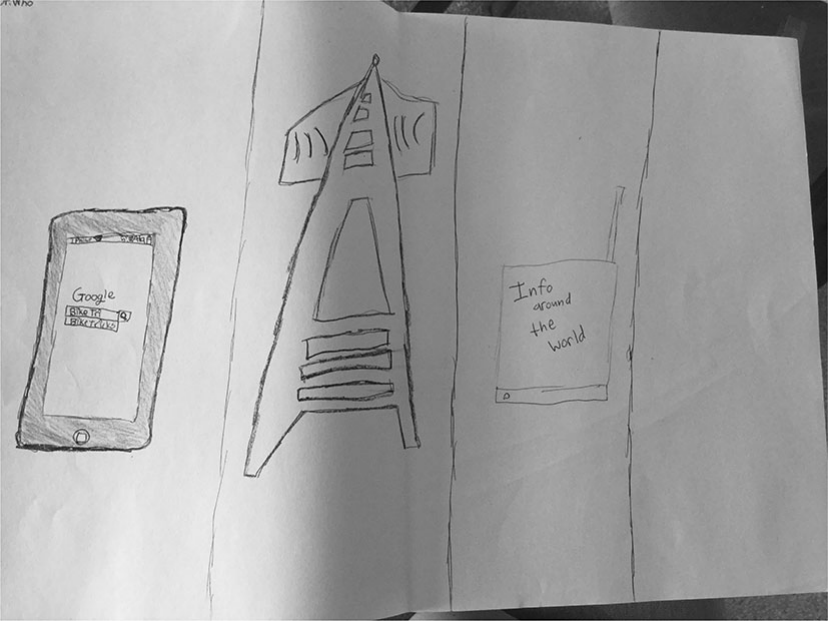
Drawing by S01

Besides S08’s drawing, Google was not primarily seen by the other students as only the computing equipment used to access it. However, several drawings did include traditional computer hardware, such as a computer monitor, keyboard, and mouse, or mobile devices, like a smart phone or tablet. It is not surprising that computing equipment would be illustrated in the students’ drawings, since these devices are the primary method through which many of them access Google. Twelve (46%) of the mental model illustrations had “Google as equipment” as either a secondary or tertiary typology entry because the student did make some mention (either verbally or in their drawing) to the equipment needed to access Google. S21’s (Fig. 9) drawing, for example, does showcase computing equipment, but his description focused more on connections. He stated, “I think when we research, there’s like an image world that it’s going around or the satellite that’s going straight to space and searching up what we’re looking up.” S01’s drawing (Fig. 10) portrays a mobile phone, an antenna, and a receiver. Although his drawing appeared to mainly focus on computing equipment, his verbal explanation went far beyond this, laying out the steps of an entire process that relied centrally on connections. He described, “You do a search up on your phone—I put in ‘bike tricks’ and then… the antenna sends it to a receiver in the Google place where they have all the people working. I put ‘info around the world’ and… [I’ll] finish with the phone having a bunch of answers, starting with the most used and the most useful.”

### Google as connections

The drawings that were categorized as “Google as connections” focused on the physical or other types of connections between Google and its users. Most of the drawings in this category focused on physical connections, such as wired or wireless connections/signals, depicting items such as antennae and satellite dishes. However, a few drawings focused more on a communication-related connection between Google and the user.

**Fig. 11:**
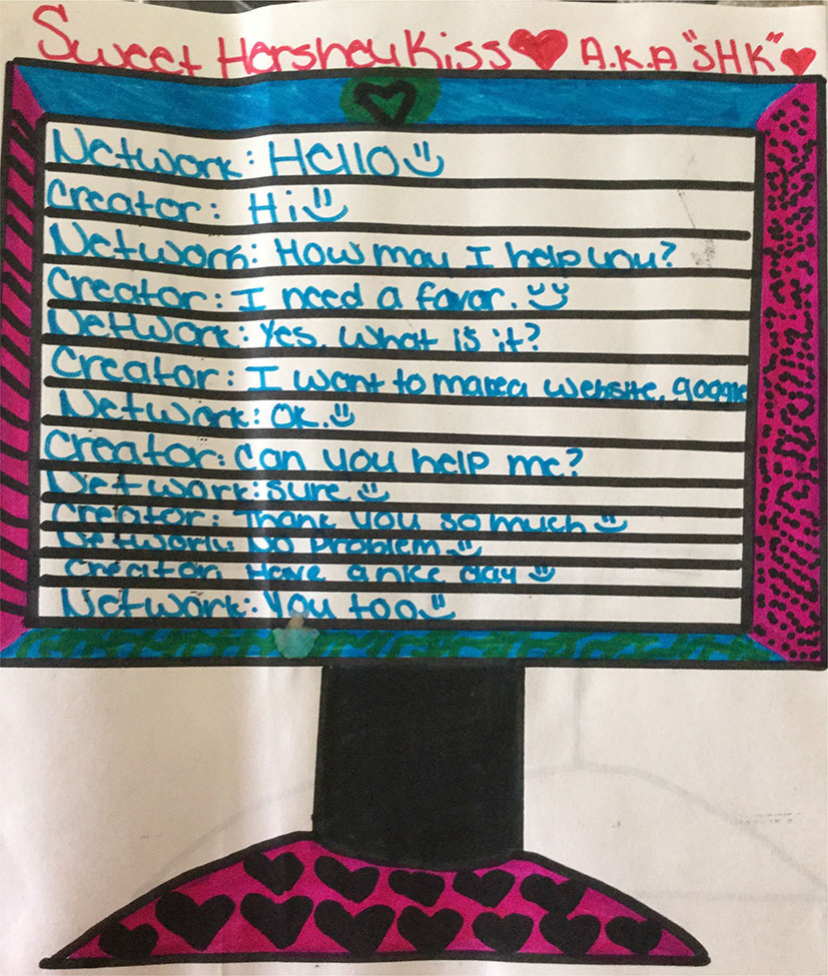
Drawing by S13

**Fig. 12:**
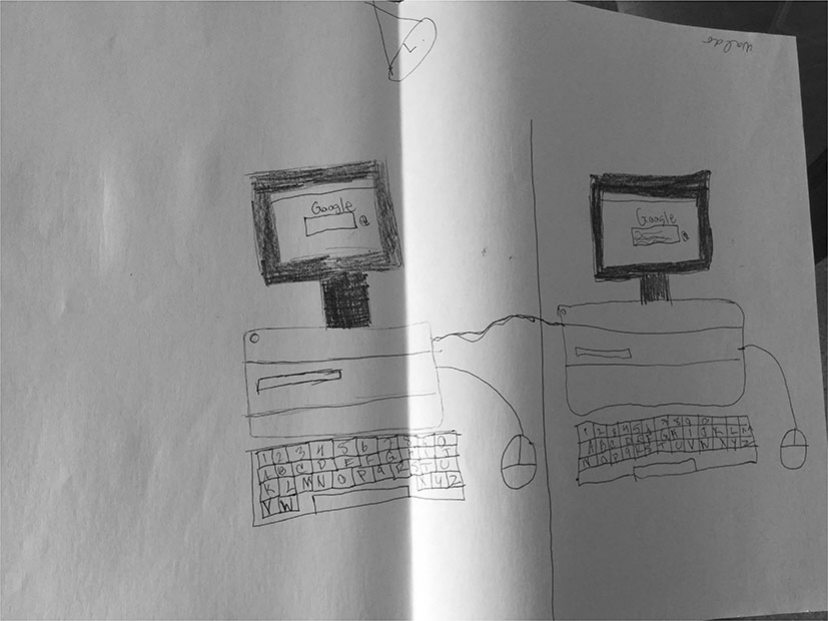
Drawing by S05

Twelve of the 26 participants (46%) referred to these connections in their drawings and/or their descriptions; however, just five (19%) participants’ drawings were assigned to this typology entry as primary. In Fig. 11, S13’s drawing shows the computer screen with a conversation between the “creator” (user) and the “network” (Google). She represents the connection between the user and Google through a conversation in which the user is asking Google to help her to make a website. S13 described, “I think Google is created by like conversation-wise. I mean everybody has to have a conversation with something.” S05 (Fig. 12) depicts Google as the connection between two computers as shown by the wires connecting them as well as the satellite on the top of the drawing that “receives the message and tells me what I need to do.” These drawings represent connections as the main focus of how Google works.

**Fig. 13:**
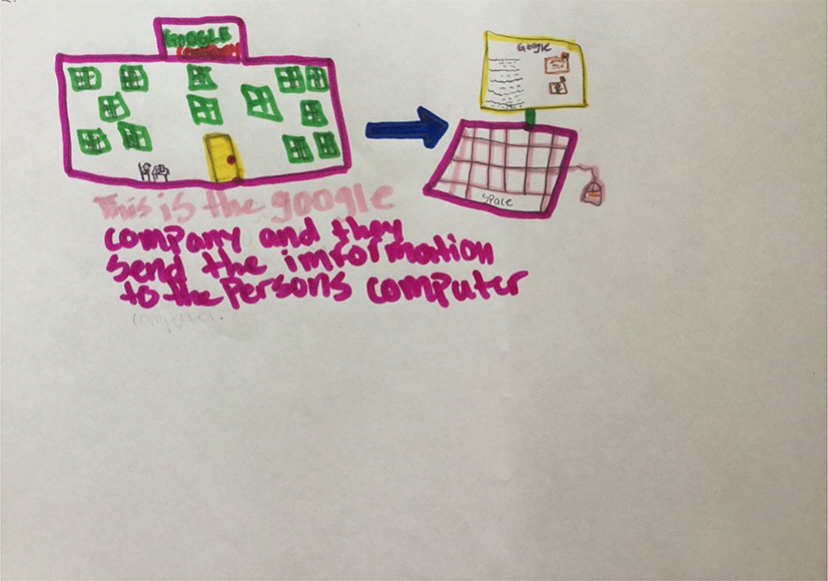
Drawing by S23

**Fig. 14:**
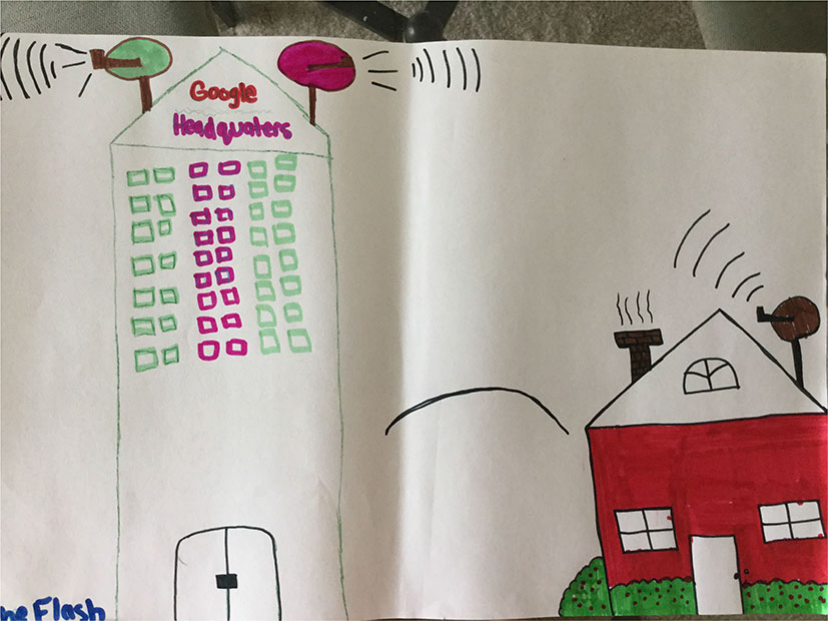
Drawing by S26

Other drawings also show Google working through various kinds of wired and/or wireless connections, but in these drawings, connections are not the main focus. Both S23 (Fig. 13) and S26 (Fig. 14) mention that Google receives a user’s search through a “signal” or a “satellite dish,” and then finds the information and transfers it back to the user. S23 described, “This is like the Google Company where all of the Google technology is and where…when you type up stuff on the computer, it automatically gets like a signal that you’re searching something and then they pick the websites that they think is best and they send it to the computer.” As both of these students’ drawings and descriptions focus on Google as connections, they focus more heavily on Google as a physical space, which is discussed in the next section.

### Google as physical space

**Fig. 15:**
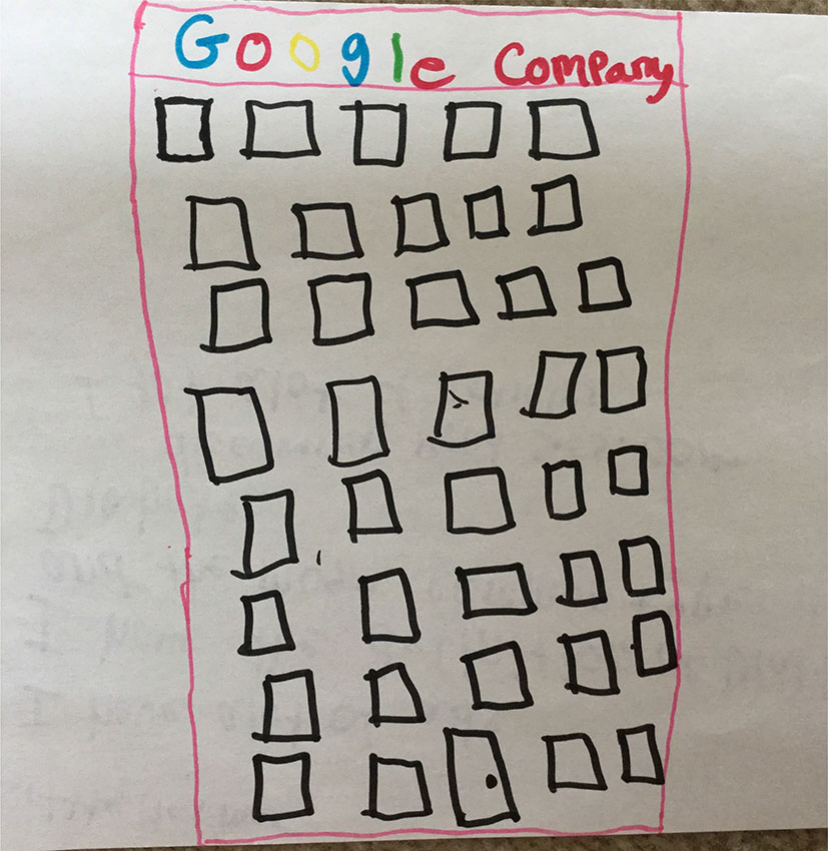
Drawing by S15

**Fig. 16:**
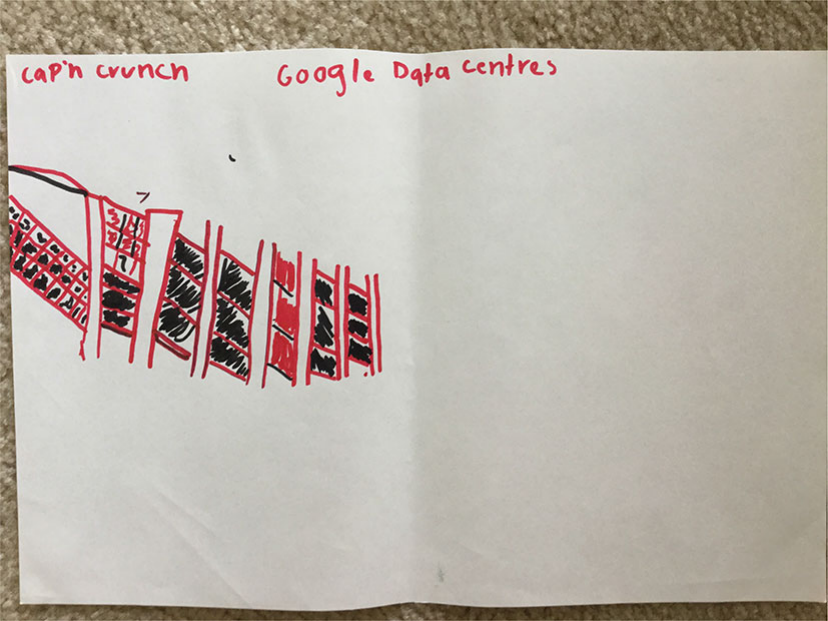
Drawing by S20

There were nine students (35%) who associated Google with a physical location. These drawings/descriptions ranged from Google being represented by a tall office building that is somehow connected to the outside world through satellites or signals to inside office spaces where “Google workers” are compiling and disseminating information to users. Four (15%) drawings were assigned to “Google as physical space” as a primary typology entry. S15 (Fig. 15), for example, depicted a very tall office building, labelling it “Google Company.” S20 (Fig. 16) drew a row of data servers, entitling his picture “Google Data Centres.” He described hearing something recently about the “top 10 places that you wish to go, but you can’t.” He recalled hearing about “mythical places” and about the Google Data Center: “They say that only a few people can enter because it’s restricted because it has all of the information… about websites that are on Google.”

**Fig. 17:**
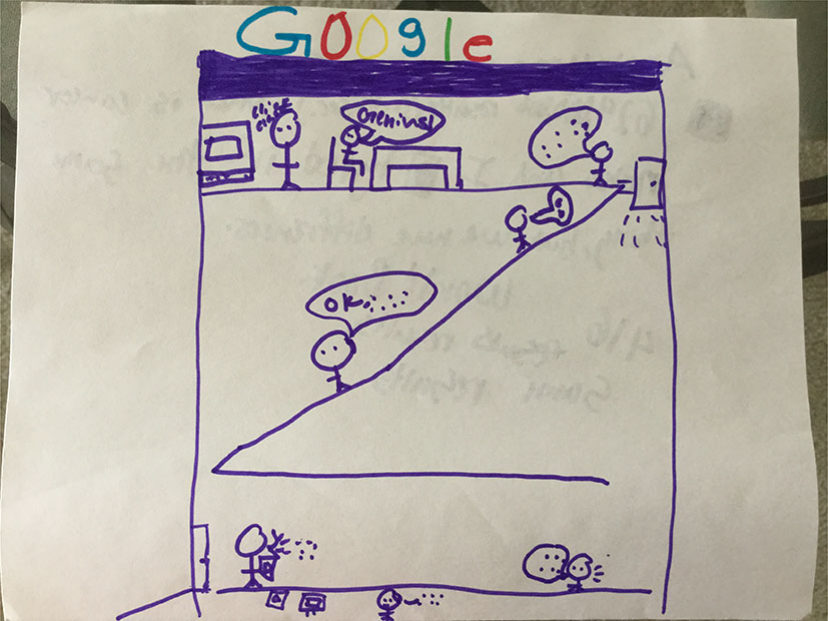
Drawing by S14

Five of the nine drawings (19%) assigned to this typology entry represented Google not only as a physical space such as a Google office building, but also as the people who work in these office buildings (“Google as people”). For example, S14 (Fig. 17) drew a picture of the Google building, but focused on the communication processes among the Google workers in trying to respond to a user’s query. He described, “You start from the bottom and then you have the people slowly going up. So you have that one person on the bottom floor who figures out this information from somebody from the outside and then… he goes up to this guy, who goes to the next guy, who goes to the next guy… There is a man who is in the basement going through things, like a social worker except for like social media and other things. And he gives it up to like his boss, who tells his boss, who tells his boss, who knows a guy who tells the boss of Google, aka the CEO, who tells him to put it all on Google.”

### Google as interface

About one-fourth of the students’ drawings and descriptions focused on what they see on the screen when they search online using the Google search engine. These drawings showed one or more detailed aspects of Google’s home and/or search results pages, such as the search bar, “I’m Feeling Lucky” button, search results count, the amount of time the search took, and images.

**Fig. 18:**
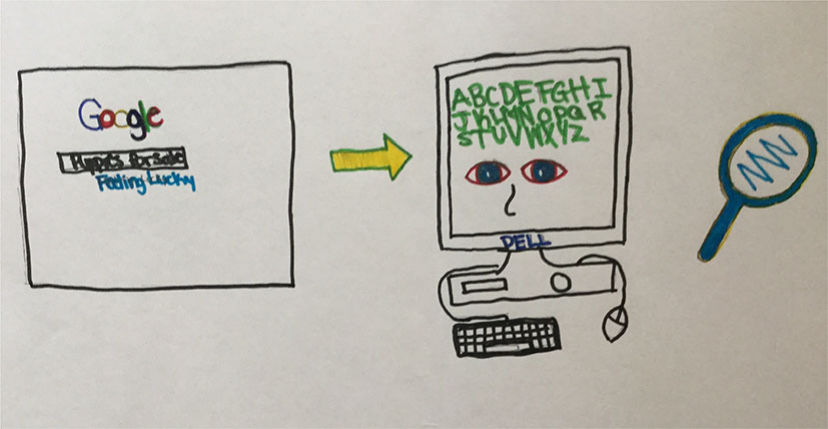
Drawing by S19

**Fig. 19:**
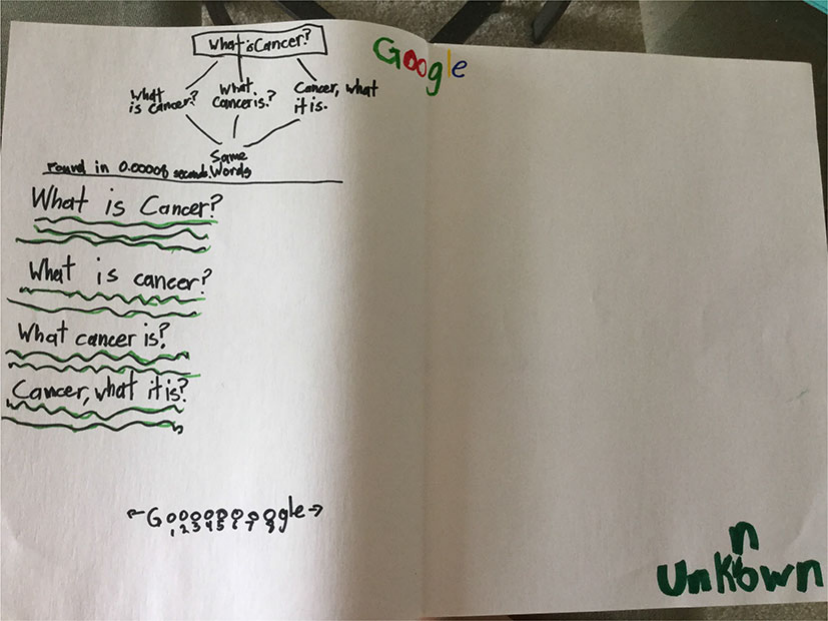
Drawing by S04

**Fig. 20:**
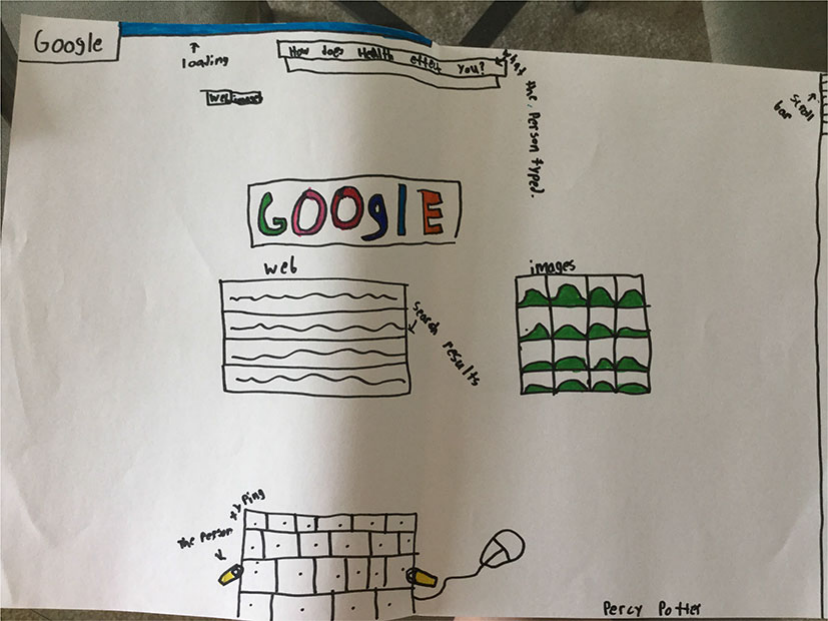
Drawing by S02

S19 (Fig. 18) drew the Google search screen, including the “I’m Feeling Lucky” button, and S04 (Fig. 19) and S02 (Fig. 20) both drew the Google search results page, highlighting different features and functionalities. All three of these students included the Google logo, replicating Google’s use of varying colors for the different letters in their name.

### Google as codes

**Fig. 21:**
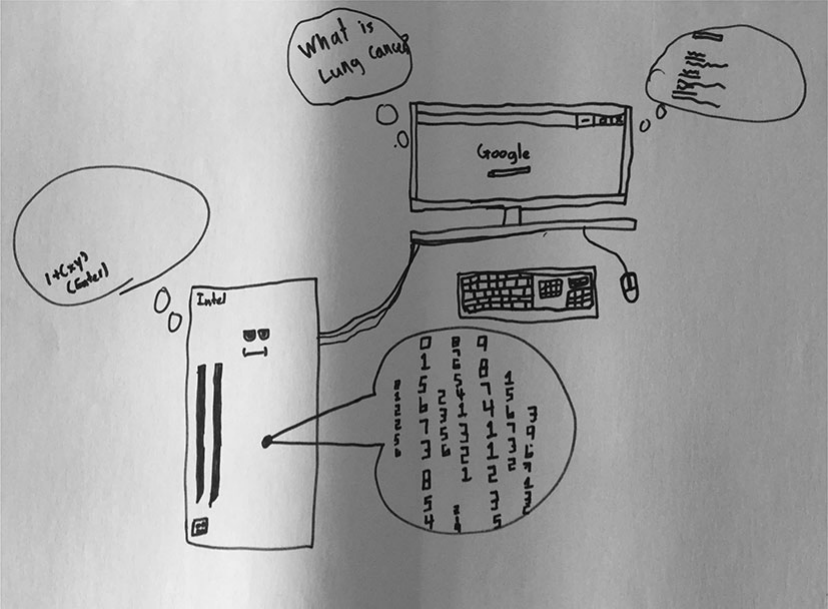
Drawing by S03

**Fig. 22:**
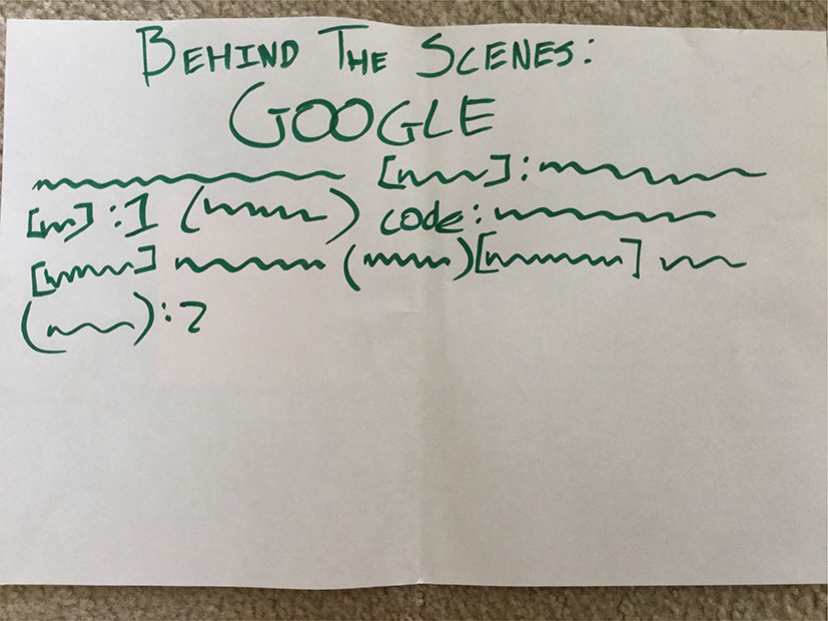
Drawing by S11

This category includes drawings that focus on codes—numeric, alphabetic, alphanumeric, or other types of symbols—that Google uses to find websites for users. “Google as codes” was the least commonly assigned entry in our typology. Just four (15%) students’ drawings were assigned to this typology entry; however, these drawings and the students’ accompanying descriptions suggest that students do have some understanding, if only superficial, of how Google actually finds websites based on user queries. S03’s (Fig. 21) drawing shows that a user typed in “What is cancer?” and the computer is depicted with the “commands” represented as computer code and a face. A monitor with Google is also shown, with the query shown in a quote bubble to the left and a series of squiggle lines in a quote bubble to the right, perhaps depicting input and output. S03 described, “The commands are in here and I made a little face for it… The person [user] types like ‘What is lung cancer?’ and then the search comes up with the answers.” S11 (Fig. 22) and S17 (Fig. 6) both make mention of Google using “codes” or “algorithms” to find information for the user, but do not elaborate on how these codes or algorithms work.

In addition to assigning each drawing to one or more typology entry, the researchers assigned one or more individual codes to each participant’s drawing. Table 5 shows the number and percentage of student drawings to which each code was applied. The most commonly occurring themes in students’ drawings were “computing equipment” (n = 20; 77%), “anthropomorphism” (n = 17; 65%), and “connections” (n = 16; 62%). More than three-quarters of participants drew some type of computer hardware in their representations of how Google works. Slightly less than two-thirds (65%) of participants’ drawings showed Google as being human in some way and/or having some human characteristic (“anthropomorphism”), and slightly fewer (62%) of the drawings depicted physical (such as cables) or wireless (such as satellites) connections that enable Google to transmit information to people. The themes of “Google worker” (n = 12; 46%) and “trust” (n = 11; 42%) were also quite common, appearing in nearly half of the student drawings.

**Table 5:** Number and percentage of drawings assigned each code

Code	Total Number of Drawings	Percentage (%) of Drawings
Computing equipment	20	76.9
Anthropomorphism	17	65.4
Connections	16	61.5
Google worker	12	46.2
Trust	11	42.3
Place	10	38.5
Query	9	34.6
Branding	7	26.9
Transparency	7	26.9
User	7	26.9
Features/functionality	5	19.2
Computer code	4	15.4
Intelligence	4	15.4
Gender balanced	3	11.5

Interestingly, nearly half (n = 11; 42%) of the students’ drawings and/or accompanying verbal descriptions evoked the concept of trust. For example, S14 described that a Google worker “gets any information that he can find, so it can be true, false, anything.” S15 similarly described Google as “a source, but there’s like a bunch of results that you can’t trust.” S17 described Google’s algorithm as a “creepy guy,” giving the user “random everything about snakes.” However, not all of these evocations were negative. S07 described: “What you don’t see is these people working really hard, going ‘hurry up!’ or ‘put the #1 best source’.” Similarly, S23 said that Google “pick[s] the websites that they think is best and send[s] it to the computer.” S01 said that his drawing would eventually show his mobile phone “having a bunch of answers, starting with the most used and the most useful.”

## Discussion

This study explored middle school students’ mental models of the search engine, Google. Participants were asked to draw how they think Google works “behind the scenes” to find websites for people and to then verbally describe their drawings. The typology and codes that emerged from our thematic content analysis of participants’ drawings and their accompanying verbal explanations of their drawings provide descriptive insights into the knowledge that youth possess on how searches are conducted by Google, and hint at the information and digital literacy instruction that they have (or have not) received. The majority of students’ drawings (58%) were deemed to fit into the primary typology entry of either *Google as people* or *Google as connections*. Students may have chosen to represent Google as a person or people because they do not have a complete understanding of how algorithms are created to work in finding search results for specific queries. Positioning a person/people behind the scenes of the computer or device screen mirrors the way people are accustomed to finding information offline—asking teachers, parents, librarians, and others questions directly and getting answers in return. Such an understanding of Google (as a person who can be expected to return a relevant response) may help to explain earlier researchers’ findings that young people may rely on surface cues in assessing the relevance of search results (Rouet et al. 2011) and may simply choose the first search result listed (Wartella et al. 2016). In depicting Google as connections, students revealed their understanding of the necessity of a technological means, whether wired or wireless, to access the information online through our devices.

One-half of the student participants included equipment (computer hardware of some kind) in their drawings and verbal descriptions. The prevalence of equipment in students’ drawings indicates their understanding of the necessity of this hardware in being able to access Google and the information and answers it provides online.

Only one-fourth of the students’ drawings depicted the Google interface and just four students included computer code or otherwise illustrated the technological functioning of Google. These findings support those of prior researchers (e.g., Holman 2011; Norman 1983; Papastergiou 2005; Rieh et al. 2010; Yan 2005, 2006, 2009; Zhang 2008a, b) who found that many participants’ mental models tend to exhibit an incomplete understanding of the system they are trying to represent. In fact, Norman’s (1983) descriptions of users’ mental models as often messy and incomplete and of users’ understandings of devices as imprecise and idiosyncratic apply to our participants’ drawings, many of whom lack experience and an encompassing understanding of Google and its various search features. However, their drawings are very creative and open a window into the ways in which they currently understand Google and the ways in which information and digital literacy instruction might be best tailored to them in order to improve the accuracy and comprehensiveness of their understanding of how Google works.

The limited understandings revealed by our participants’ drawings suggest two primary implications of this research. First, as educators, we will need to revisit how we teach the concept of search engines and the search process to youth. Second, there is a need for search engine developers and search engine interface designers to make Google’s actual search processes (including their PageRank algorithm and their Search Engine Optimization processes) more transparent and trustworthy to users, particularly to younger users. One way in which this might be achieved is to include explanatory symbols next to each item in a search engine results list. For example, one set of symbols could be used to denote the quantity of websites that link to the particular site, another to denote the average quality of these websites, and yet another to denote the type of organization (e.g., a non-profit organization, a University, or a hospital) that authored the listed site.

A close examination of the *Standards for the 21st Century Learner in Action* (American Association of School Libraries 2009), which includes the primary guidelines for information and digital literacy in the United States, and *Web Literacy,* developed by the Mozilla Foundation (Mozilla Learning 2016), reveal that there is much emphasis in learning how to develop keywords and questions to find the information needed on the open Web, but no emphasis given to examining how search engines actually work. Learning how to select the best keywords and develop effective queries is no doubt useful, but actually helping youth understand how search engines function may enable youth to be better able to come up with keywords and formulate questions that will produce more relevant and useful search results and help youth in choosing which search results are likely to provide the most credible and relevant information for their inquiries. While youth do not seek information solely from the open Web but are encouraged to use databases where the use of effective queries is essential, it is vital that youth are informed about how search engines work. A recent study conducted by the research team to develop a digital health literacy skills inventory with a particular focus on online health information seeking incorporated “Understand how search engines work (i.e., hits, order of search results, snippets, inclusion/placement of ads, etc.)” as a skill that must be mastered (Subramaniam et al. 2015b). Prior research (e.g., Dinet and Kitajima 2011) has found a correlation between people’s mental models of a system and their ability to make effective use of that system. Without an accurate and comprehensive understanding of the ways in which Google functions, youth are at a distinct disadvantage in trying to interact with the Google interface, evaluate the results returned, and ultimately find relevant and trustworthy information that will be useful for them.

This study revealed that a large number of youth (65%) refer to Google as an individual person or a group of workers, and/or invoke the concept of trust (or mistrust) in Google as a source of information (42%) in their drawings and accompanying verbal descriptions. While this anthropomorphism is a pretty common finding in relation to children’s and adults’ conceptions of computers and search engines (see, for example, Hendry and Efthimiadis 2008; Proudfoot 2011; Rücker and Pinkwart 2016), the focus on the concept of trust in the “people” behind the search engines when describing how search engines work is a novel finding. This provides an impetus for search engine developers to more clearly and more readily convey to users how search results were selected for inclusion in the search results list and how they were sorted in order to better educate users (in this case, youth) on how they can effectively and efficiently assess the credibility of search results, given the nature of their queries. Previous studies (e.g., Eastin 2008; Flanagin and Metzger 2008; Subramaniam et al. 2015a) have shown that youth have tremendous difficulty in assessing the credibility of search results and of the information they obtain online. Thus, comprehending how the search engine generated the results list may actually help youth to make wise decisions in their selection of information and enable them to form effective heuristics for assessing the credibility of open Web information sources.

While this study begins the conversation on youths’ mental models of how search engines work, the study itself has several limitations. First, as other researchers have found (e.g., Barrett and Bridson 1983; Light 1985; Panagiotaki et al. 2009), our participants’ drawings may have been influenced by the instructions that we provided in regard to both the drawing activity and the subsequent verbal explanations of their drawings that we requested. Additionally, as participants shared their drawings in groups, some participants’ verbal explanations of their drawings may have been influenced by the explanations provided by the students who went before them.

Second, our participants may have had inadequate manual dexterity skills and/or verbal/written capabilities to accurately draw or describe their mental models of the Google search process, as had been discussed by Marhan et al. (2012). As mentioned in the results section above, some of these youth did not provide adequate written/verbal descriptions to support their drawings, exclusively focused on a single aspect of the entire search process (e.g., the communication process of Google workers), or drew a mental model that did not match their written/verbal descriptions. Future research studies aiming to elicit youths’ mental models can include a series of examinations of their mental models through contextual drawings and verbal/written explanations within the context of a specific search that they actually initiate. This will allow juxtaposing their descriptions across multiple searches that they conduct rather than a single opportunity to infer their mental models. This will also eliminate previous concerns in the literature whereby participants may feel pressured to come up with a mental model simply because they are asked to do so by researchers (Norman 1983; Richardson et al. 1994).

Third, although we are convinced that the participants in *HackHealth* all have very little exposure and background in computing and new media literacy based on descriptions provided by the school librarians about their information and digital literacy skills instruction and our own firsthand observations through the *HackHealth* program, we were unable to do a cross-comparative analysis of how their previous experience in computing or digital literacy development (if any) impacted their mental models of how Google works. Although it is likely that our older participants had more exposure to Google than our younger participants, we did not attempt to systematically assess this variable. As a result, we can only rely on our observations of participants’ interactions with Google and on our informal inquiries into this matter with the participating school librarians. Future research should uncover the impacts that relevant computing or digital media instruction in schools has on the development of youths’ mental models.

Fourth, our study was able to provide a typology of the mental models of the Google search engine as conveyed by the youth we worked with in the *HackHealth* after-school program. Further studies will need to be conducted to refine the typology with a larger sample of youth in this age group who come from different types of socioeconomic, educational, and other demographic backgrounds. Additionally, subsequent studies will also need to investigate the factors contributing to inaccurate and/or uncomprehensive mental models and track the development of mental models before and after instruction is given on how search engines work.

## Conclusion

This study provides a window into the many views that youth have on how Google works. In recent years, there has been a growing number of studies that investigate how youth interact with computing systems and digital media, particularly how they seek information on the open Web, assess the credibility of information, and/or confirm/reject their biases as they decide which information to use (e.g., Agosto 2002a, b; Eastin 2008; Flanagin and Metzger 2008; Francke et al. 2011; Gasser et al. 2012; Hansen et al. 2003; Shenton and Dixon 2004; St. Jean et al. 2015; Subramaniam et al. 2015a, b). Future research is needed to explore the existence/nature of the relationship between young people’s mental models of search engines and the heuristics they rely on when interacting with them and assessing the credibility of online information.
